# Research on oral microbiota of monozygotic twins with discordant caries experience - *in vitro* and *in vivo* study

**DOI:** 10.1038/s41598-018-25636-w

**Published:** 2018-05-08

**Authors:** Hongle Wu, Benhua Zeng, Bolei Li, Biao Ren, Jianhua Zhao, Mingyun Li, Xian Peng, Mingye Feng, Jiyao Li, Hong Wei, Lei Cheng, Xuedong Zhou

**Affiliations:** 1 State Key Laboratory of Oral Diseases, Sichuan University, 610041 Chengdu, China; 2 Department of Cariology and Endodontics, West China Hospital of Stomatology, Sichuan University, Chengdu, 610041 China; 3 National Clinical Research Center for Oral Diseases, Sichuan University, Chengdu, 610041 China; 4 Department of Laboratory Animal Science, College of Basic Medical Sciences, Third Military Medical University, Chongqing, 400038 China; 5 Shanghai Majorbio Bio-pharm Technology Co., Ltd, Shanghai, 201203 China; 6 Department of Immuno-Oncology, Beckman Research Institute, City of Hope Comprehensive Cancer Center, Duarte, CA 91010 USA

## Abstract

Oral microbiome is potentially correlated with many diseases, such as dental caries, periodontitis, oral cancer and some systemic diseases. Twin model, as an effective method for studying human microbiota, is widely used in research of relationship between oral microbiota and dental caries. However, there were few researches focusing on caries discordant twins. In this study, *in vitro* assays were conducted combined with 16S rRNA sequencing analysis on oral microbiota sampled from twins who presented discordant caries experience and mice model was developed as well. Results showed that oral microbiota from caries-active twin possessed higher metabolic activity and produced more lactic production. 16S rRNA sequencing analysis showed that more than 80% of family taxa could be transferred into gnotobiotic-mice. Key caries-associated genera were significantly different between twins and the same difference in genus level could be found in mice as well (p < 0.05). This study suggested that oral microbiota of twins could be distinguished from each other despite the similarities in genetic make-up, living environment, and lifestyle. The difference in microbiota was applied to develop a mice model which may facilitate the investigation of core microbiota of dental caries.

## Introduction

Dental caries, a multi-factors related disease, is a major factor of irreversible tooth decay and will in further lead to pulpitis, severe toothache, and chronic infections^1^. The etiology and related risk factors of dental caries have been a hot topic for a long period of time. The microbial factors are known as indispensable causes of the onset and progress of dental caries. Recently, core microbiota was considered to play important roles in the development of diseases, such as obesity and dental peri-implantitis^2,3^. Previous studies claimed that core microbiota was one of the most important elements in dental caries development^4–6^. More efforts should be put on exploring how core microbiota determines the progress of dental caries via *in vitro* and *in vivo* experiments. Therefore, an effective and accurate model is highly in need. The existing *in vitro* mono- or multi- species biofilm model^7,8^ or saliva biofilm model^9^ were utilized to simulate cariogenesis environment. *Streptococcus mutans* biofilm is one of the examples which contributed to the development of anti-caries material^8,10^. In our previous studies, we found that the growth media, not the substrates, have significant effects on the microbial community of saliva-derived biofilm *in vitro*^11^. However, *in vitro* model was unable to completely restore and delegate inner oral environment and microbiota due to the pH, temperature, enzymes and so forth. It can only reflect the condition of biofilm in exact situation.

Furthermore, there are other factors that can contribute to onset of dental caries, for instance, speed of salivary flow, saliva composition, fluoride exposure^12,13^, dietary habits, physical status, genetic factors and living conditions^14–16^. The common *in vitro* model can hardly take these factors into account. Thus, *in vivo* animal model is likely to be a better model than *in vitro* to imitate a relatively similar oral environment of human being. In our previous study, human oral microbiota-associated mouse model had developed via transplanting human saliva to mice. It successfully reconstructed human oral microbiota in gnotobiotic mice (results under review) and it can technically be used.

Biotechnology and bioinformatics methods develop rapidly in recent years, which stimulate the establishment of a better understanding of both cultured and un-cultured bacteria and the microbiota-related diseases. At the same time, those methods enable the determination of the composition and possible functions of microbiota in specific niches^17–19^. These brand-new technologies refresh the previous views on microbes in human body and relevant researches emerge to the public in an endless stream. A suitable model can possibly enhance the efficiency along with these technologies. For this purpose, a more effective research model is supposed to be established. Combining the utilization of biotechnology and bioinformatics, the highly precise results in human microbiome researches could be possessed.

Twins research involves comparisons of similarity or discrepancy between monozygotic twins or between monozygotic and dizygotic twins^20–22^. According to these comparisons, researchers can estimate the importance of genetic or environmental factors that influence the variation of human physical characteristics^22–24^. For example, Townsend *et al*. emphasized the value of twin studies in clarifying the effects of genetic factors on common dental problems, such as dental caries, periodontal diseases and malocclusion^25^. Because of the one hundred percent identical genes and comparable living conditions and lifestyles, monozygotic twins are supposed to own the same body features. Nonetheless, the twins discordant for obesity have been recruited in a research of gut microbiota^26^ implying the influence of the gut microbiota on the development of obesity using the germ-free mice model.

Papapostolou *et al*. reported that twins have similar experience of dental caries^27^. Nevertheless, it was observed that identical twins with the same diet, lifestyle and living environment can keep different DMFT. Therefore, twins with discordant caries experience might be a useful model for investigating the core microbiota of dental caries. Besides, it is important to mimic the different oral microbiota derived from the monozygotic twins with different dental caries experience. So far, there is no study investigating the difference of caries experience between those monozygotic twins. Therefore, based on the *in vitro* biofilm model and oral microbiota associated mice model, the present research is aimed to compare the oral microbiota between monozygotic twins with discordant caries.

## Results

### Relevant characteristics of the twins

In this study, one pair of 23-year-old female monozygotic twins were recruited. The physical conditions were recorded and showed in Table 1. Both participants were born via cesarean. They lived together and had never been apart. Their eating habits were quite similar and both of them had preference to sweet foods. BMI index were 19.19 (the elder sister) and 18.82 (the younger sister) respectively when they took part in the research. As for the results of oral examination, both subjects possessed 24 teeth and the DMFT index of the elder twin was 0 and the younger one was 4. (Table 1).

**Table 1:** General characteristics of the twins recruited in this study.

	The elder	The younger
Gender	Female	Female
Age	23	23
BMI	19.19	18.82
Smoking history	No	No
Chronic disease	No	No
Oral hygiene habit	Brushing twice a day, about 3 minutes	Brushing twice a day, about 3 minutes
Food preference	Sweet and hot food	Sweet and hot food
DMFT	0	4
Salivary buffering capacity	Medium	Medium

### Results of *in vitro* experiments of oral microbiota

Saliva and plaque samples were used to form biofilm in SHI medium^28^ on hydroxyapatite (HA) disks. Then, the biofilms were subjected to 3-(4,5-Dimethylthiazol-2-yl)-2,5-diphenyltetrazolium bromide assays (MTT) and lactic acid measurements.

**Figure 1:**
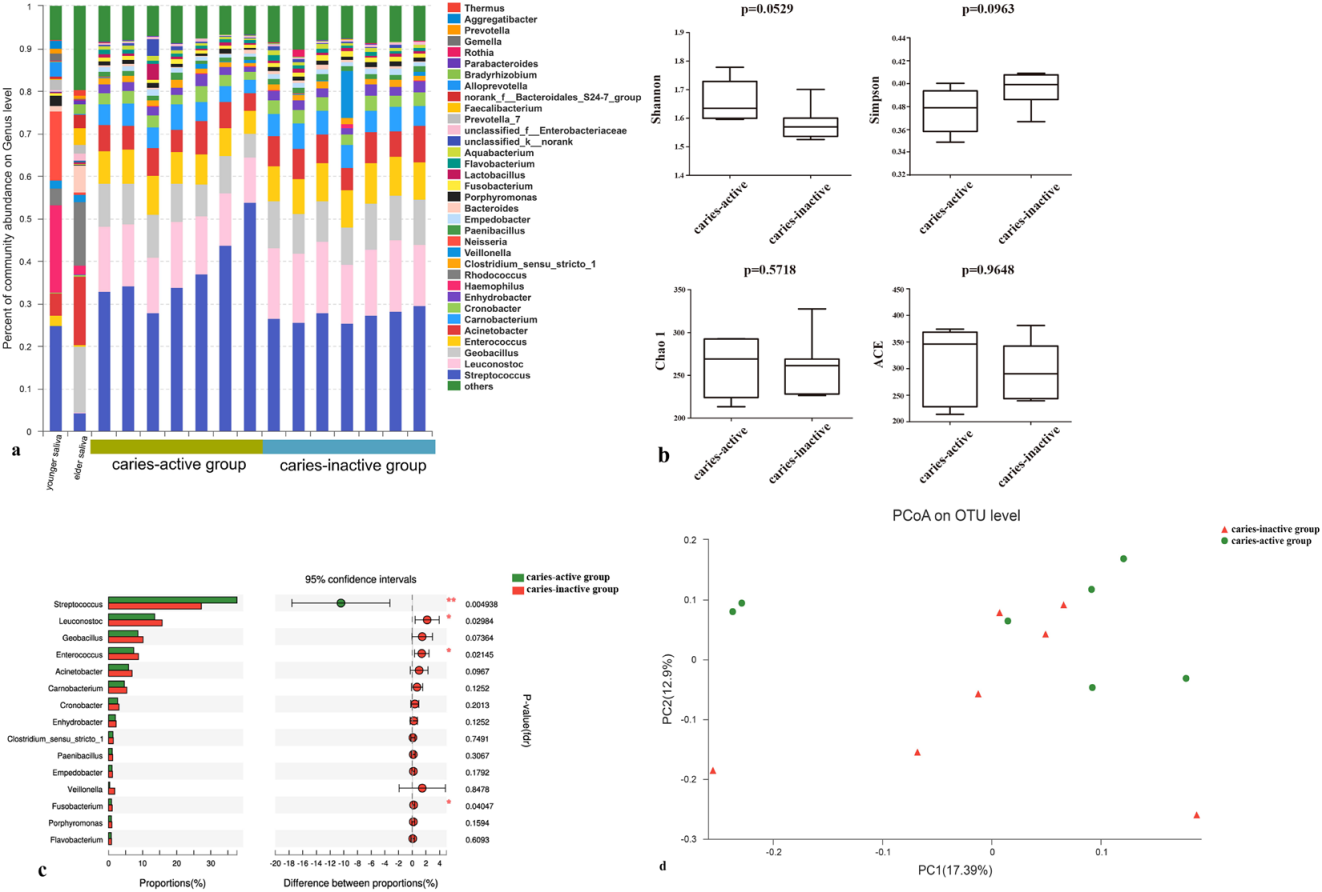
(**a**) Results of MTT test. Each value is the mean ± SD (n = 6). Values with stars on top are significantly different from each other. (*p* < 0.001; t-test). (**b**) Result of lactic acid production test.

The MTT assay results (mean ± SD; n = 6) illustrated that saliva biofilm of the younger twin on hydroxyapatite(HA) specimen had almost 3 folds greater absorbance than the elder twin (p < 0.05) (Fig. 1a). Results of lactic acid productions of biofilm were plotted in Fig. 1b. Similarly, biofilm of saliva formed on HA specimen of the younger twin produced more lactic acid than the elder twin (*p* < 0.05). To the summary, the oral microbiota of the younger twin, who got higher DMFT, metabolized more absorbable nutrition and produced more lactic acid, which was a risk factor of caries. The higher metabolic viability and more lactic acid production may contribute to the formation of dental decay on host.

### 16S rRNA sequencing analysis of twins’ saliva and dental plaque

**Figure 2:**
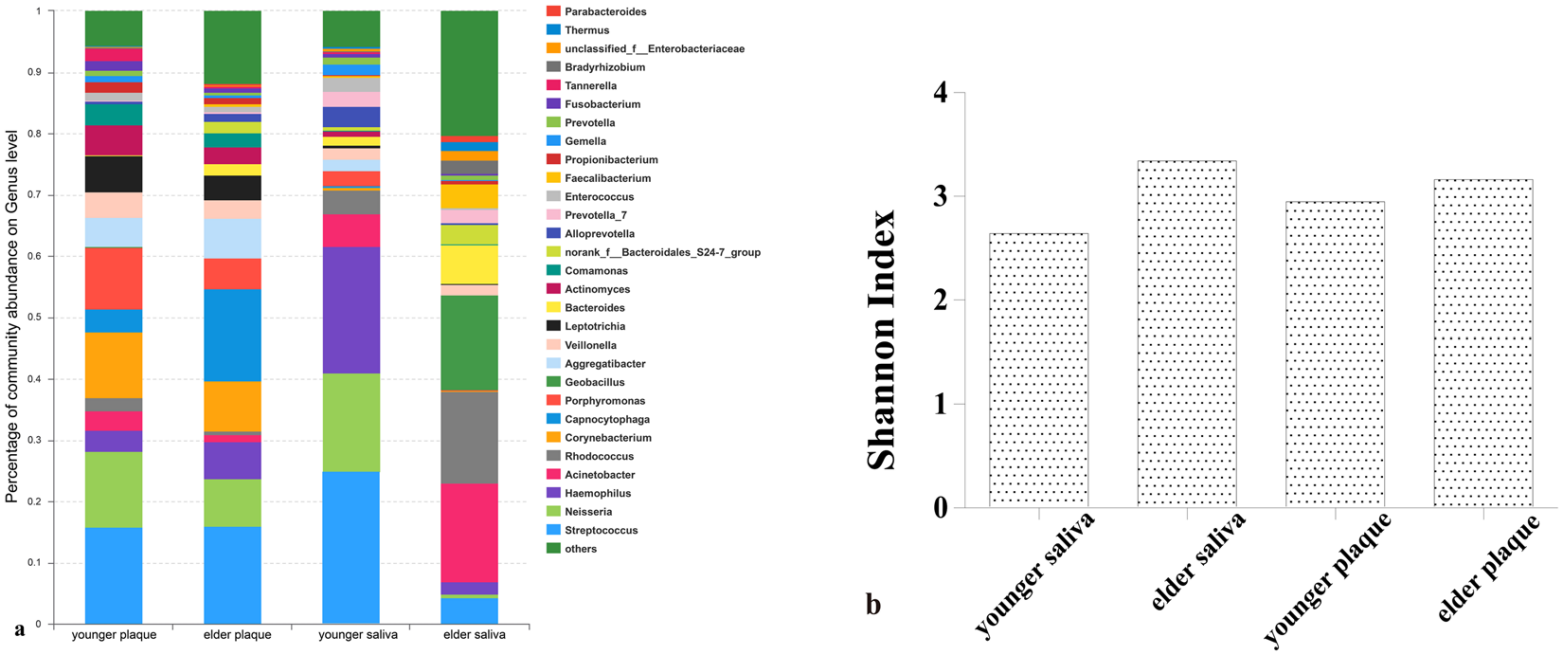
Composition and diversity of bacterial community in saliva and dental plaque samples of the twins. (**a**) Abundance of bacteria of the twins’ saliva and plaque samples at genus level. (**b**) Diversity of bacterial community from the host saliva and dental plaque samples.

The microbial communities in saliva and dental plaque of the discordant caries experienced-twins were characterized through 16S rRNA sequencing analysis and the differences of richness, diversity and relative abundances of each taxon were determined. In total, 16 phyla, 27 classes, 63 orders, 116 families, 294 genera, 484 species and 688 OTUs were detected from samples of the two participants. As for diversity, the Shannon index showed that microbial diversities were higher in both plaque and saliva samples of the elder than the younger twin (Fig. 2b).

In 688 OTUs, 117 of which were detected in all the 4 samples, and 255 and 172 OTUs were shared by twins in saliva and plaque respectively (Supplementary Figure S1). The shared OTUs constituted 69.7% and 54.1% of all OTUs in the younger and the elder twin saliva samples, while that in their plaque samples occupied 70.5% and 39.8% of total OTUs. Though the community abundances were similar in both participants, the composition has some differences between twins.

In dental plaque samples, the dominant phyla were *Proteobacteria, Firmicutes* and *Bacteroidetes*, while in saliva samples, *Firmicute* and *Proteobacteria* were the most abundant phyla (both presented greater than 50%). At genus level, *Streptococcus* and *Neisseria* were the majority in saliva and plaque (Fig. 2a) except saliva of the elder which was predominated by *Acinetobacter* and *Rhodococcus*. The intra-pair twins‘ discrepancy of oral microbiota was smaller than unrelated individuals, which was proved by existing studies^29^. However, in this study, the difference of salivary microbiota was remarkable. Additionally, the proportions of the predominant taxa at the genus level were also compared. On one hand, there was a greater percentage of *Streptococcus, Neisseria, Haemophilus* and *Veillonella* in saliva of the younger sister, while the higher percentage of *Acinetobacter, Rhodococcus* and *Geobacillus* were found in saliva of the elder sister. On the other hand, in plaque community, more *Streptococcus, Capnocytophaga* and *Lysinibacillus* presented in the elder twin but there were more *Neisseria, Corynebacterium, Porphyromonas* and *Leptotrichia* observed in the younger sister.

In summary, from the 16S rRNA sequencing analysis, it was certified that there were several differences existing in microbiota of dental plaque and saliva of the twins the differences in the microbial diversity, composition of microbiota and the phyla colonized in saliva and dental plaque was observed in present study. The future research on the association between discrepancies and discordant caries experience is in need, however, whether those discrepancies contributed to the discordant caries experience between twins deserve further exploration.

### Results of gnotobiotic mice trial

The saliva (stored in −80 °C before experiment) of the monozygotic twins were gavaged into recipient mice (2 groups, n = 7) respectively. After 5 weeks, 14 saliva samples were collected from each mice group and 16S rRNA gene amplicons were sequenced.

#### Characteristics of saliva microbiota in mice oral cavit**y**

**Figure 3:**
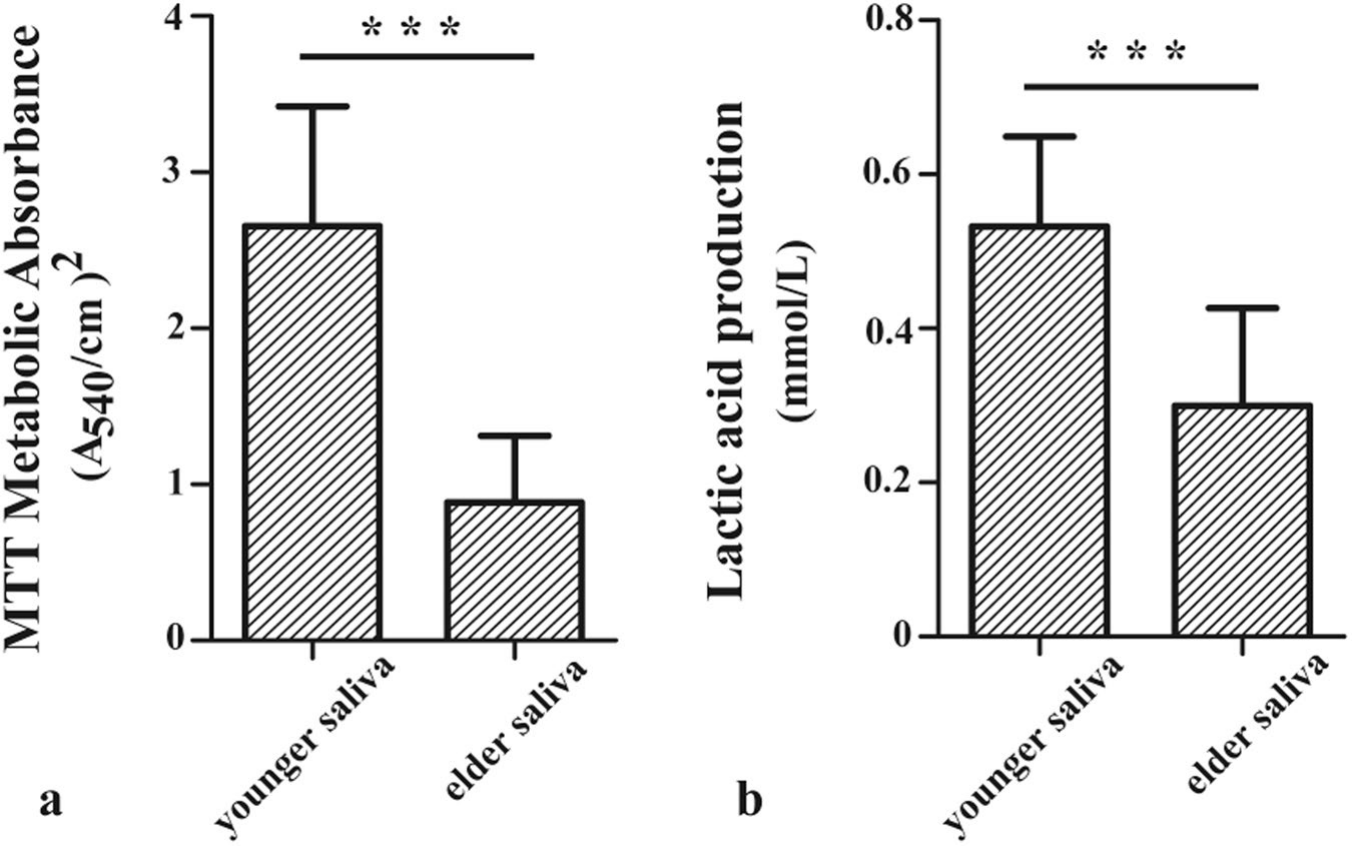
16S rRNA sequence analysis of two groups of host saliva-transplanted mice; the caries-active group received saliva from the younger sister with caries while the caries-inactive group received saliva from the elder sister without caries. (**a**) Abundance of microorganism in twins and mice saliva. (**b**) Alpha diversity comparison in Shannon, Simpson, Chao 1 and ACE index (*p* > 0.05; t-test) of groups of mice. (**c**) Top 15 different genera-level taxa in mice oral microbiota with different host saliva samples. (* represented for significant difference and ** stand for extremely different between two groups, Wilcoxon rank-sum test). (**d**) PCoA result of comparison between two group of mice.

Saliva microbiota of two groups of mice which received saliva from the caries active twin and the caries inactive twin were analyzed. Similar composition of saliva microbiota was developed by mice received distinct saliva. All samples contained considerable percentage of *Firmicutes* at phylum level, *Proteobacteria* and *Bacteroidetes* also showed high abundance. At genus level, *Streptococcus, Leuconostoc, Geobacillus* and *Enterococcus* were very common genera in mice oral cavity (Fig. 3a). *Streptococcus, Leuconostoc, Geobacillus, Enterococcus* and *Acinetobacter* were the major genera and ranked the top 5 genera in both groups, most of which were common in human oral microbial community. Nevertheless, there were some variations in abundance of genus-level taxa between two groups. More *Streptococcus* colonized in mice of caries-active group (mice who received saliva from the younger twin) than inactive group (the abundance was 37.77% vs. 26.9%), and the difference was significant (*p* < 0.05). As for caries-inactive group (mice who received saliva from the elder sister), there were higher percentage of *Leuconostoc* (*p* < 0.05), *Fusobacterium* (*p* < 0.05) and *Enterococcus* (*p* < 0.05). In addition, Geobacillus and Acinetobacter were also more abundant in caries-inactive group abundances were not significantly different (p > 0.05) (Fig. 3c).

Moreover, Shannon index indicated that caries-active group was higher in diversity than caries-inactive group, but the difference was not significant (*p* > 0.05). Similarly, Simpson, ACE and Chao1 indices were not significantly varied between two groups (*p* > 0.05) (Fig. 3b).

As for the beta diversity, the PCoA was conducted and the result showed that two groups of mice possessed similar saliva oral microbiota (Fig. 3d).

#### Comparison of hosts oral microbiota and mice saliva microbiota

The saliva microbiota of mice and hosts were compared. In general, we successfully transplanted human oral microbiota into mice oral cavity. Previous researches had developed humanized mice model and transplanted 88% of genus-level taxa and 11 of 12 bacterial classes of donor gut microbiota into mice gut^30^. The transplantation rates in the present study reached 88.4% and 81.6% at family level in caries-active group and caries-inactive group respectively, which was consistent to our previous study. Besides, 90% and 80.9% of bacterial orders were transplanted into mice and at the class level, the transplantation rates were 90% and 86.4% in caries-active group and caries-inactive group. The mice model that recruited twins was successfully constructed, and it was able to receive the majority of microbiota from the donors.

Meanwhile, we found that recipient mice could not only presented the oral microbiota of the donors but also displayed the difference of oral microbiota between the donors. In this study, the genera, occupying higher than 1% of total microbiota, whose quantitative difference between two donors was higher than 10% were selected. Meanwhile, the genera occupying over 0.1% but less than 1% and the quantitative differences being more than 0.3% between two individuals were selected (Supplementary Table S1). Genera *Streptococcus, Haemophilus, Acinetobacter, Rhodococcus* and *Neisseria* and so on were included. In comparison to the previous study proved that *Streptococcus, Prevotella, Leptotrichia, Veillonella* and *Fusobacterium* were correlated with onset and progress of caries^18^, and we found significant difference between the abundance of *Streptococcus, Prevotella, Leptotrichia* and *Fusobacterium* of the twins in the present study. 3 of these taxa also showed obvious difference in the mice oral cavity including *Streptococcus, Prevotella* and *Fusobacterium*. 75% of key taxa variations in host were successfully transferred into mice and they could reflect the difference between oral microbiota of the monozygotic twins. In addition, *Veillonella* showed no difference in both twins‘ saliva and mice saliva. The successful transplantation of difference in caries-correlated taxa indicates the possibility of investigation of caries-associated microbiota via our mice model utilized caries discordant twin pairs.

## Discussion

In this research, the involved monozygotic twins possessed theoretically same gene, similar living environment as well as dietary habits according to the questionnaires. However, they had discordant caries experiences. The younger sister underwent dental treatment due to caries and subsequently diagnosed with caries in mandibular molars. On the contrary, the elder sister never had caries. In this study, emphasis was put on the bacterial factor which was well known to have great impact on progress of dental caries. Higher metabolic viability and more lactic acid production of biofilm formed by saliva of the caries-active twin were observed. Although *in vitro* test cannot completely mimic the real environment in host, due to the improvement of culture media, the composition and diversity of *in vitro* saliva-derived biofilm presented higher similarity to host oral microbiota than previous studies^11,28^, indicating the reliability of *in vitro* experiment. However, in recent decades, non-cultured technology was becoming mainstream gradually, a mice model was taken into consideration using caries discordant twin based on the previous work about gut microbiota transplantation^26,30^.

Differences of the twins‘ oral microbiota were detected through 16S rRNA sequencing. Teng *et al*. reported that there existed 20 species of bacteria that played an important role in the prediction of early-childhood caries and might have strong correlation with onset of caries^18^. In our previous research, 5 species were selected, namely *Streptococcus*, *Prevotella, Leptotrichia, Fusobacterium* and *Veillonella*. Results showed that the multi-species microbial biofilm containing these 5 genera had greater ability of demineralization, contributing to the accelerated the caries development. There were several potential caries-associated bacteria found in caries-active host including *Streptococcus*, *Prevotella, Leptotrichia* and *Fusobacterium* except *Veillonella*, which were different from previous researches^18^. The possible reason may be that former study tried to found the predicative bacterial community of early childhood caries while adult participants were recruited in this study. However, whether this disparity suggesting *Veillonella* playing different roles in childhood and adult caries warranted further examinations and verifications. At the same time, there was always a debate on Veillonella about its role in dental caries. Previous Existing researches reported that it was had highly correlatedion with caries^31,32^, but a latest study suggested presented a controversy result^33^ and researchers pointed out that Veillonella may be related contribute to caries-free state. Even though, there was a consensus reported that the Veillonella was a health-associated genus which can be utilized to prevent disease in the future^34^. This debate remained to be settled and our analysis will be a key point of later study on how Veillonella can influence the dental caries. The association between Veillonella and dental caries requires confirmation of further study. Our findings could be an entry point in future exploration of how Veillonella can influence the dental caries.

According to the results of 16S rRNA sequencing, there was higher percentage of Streptococcus in both saliva and plaque from caries-active participant than another one. We all have known that Streptococcus is a potential caries-related bacterium, a lot of research have tested its capacity of acid production, duration of acid and ability to adhere to tooth surface. Nevertheless, the reason behind monozygotic twins with different caries experience is not clearly yet. The role that Streptococcus plays warrant further investigation.

In this study, human oral microbiota related mice model not only successfully expressed microbiota transplanted from human oral saliva, but also copied variations of key potential caries-related taxa of the caries discordant twins. Gnotobiotic mice model is of increasing popularity in the investigation of the relationship between gut microbiome and diseases in replacement of human body^35^. Nevertheless, gnotobiotic mice model is barely used in oral microbiome research, so, we are trying to use this mice model in the study of possible mechanism of discordance in the oral microbiota from the caries-discordant twins. Furthermore, the core microbiota of dental caries is likely to be a new therapeutic target for preventing and treating dental caries.

In conclusion, this research explored the difference of metabolic activity and lactic acid production of the discordant caries experience in monozygotic twins and this kind of twins’ oral microbiota was firstly utilized to construct humanized mice model, in which the characteristics of those recipient mice were analyzed. However, due to the small sample size, the different metabolic activity and colonization of oral microbiota between twins need further confirmation. And the modifications and insightful investigations are in need to better mice model.

## Materials and Methods

### Ethical considerations

The study was conducted in accordance with the Declaration of Helsinki, the policy of Sichuan University and West China School of Stomatology, and the protocol was approved by the Ethical Committee of West China School of Stomatology, Sichuan University (Chengdu, China) (Project identification code: WCHSIRB-D-2016-070, approval date: 03/03/2016). The written informed consent was obtained from each participant upon admission to oral examination, photos and samples.

### Samples Characterizations

The samples consisted of 1 pair of twins who were 23-year-old females, and informed consents for inclusion were obtained from participants. Assessment of oral health showed that the elder one had zero DMFT, while the younger sister has 4 DMFT. None of them had systemic diseases and antibiotic usage in last 3 months. They lived together since they were born and had same oral hygiene behaviors.

### Twins saliva and dental plaque collection

Saliva was collected from the adult twin donors, and participants did not use antibiotics within the past three months. The donors were demanded not to brush teeth for 24 h before collection and abstained from food/drink intake for at least 2 hours prior to donating saliva. Stimulated saliva was collected during parafilm chewing and kept in ice. Saliva samples were centrifuged at 2600 g for 10 min to remove large debris and eukaryotic cells, then each sample was diluted in sterile glycerol to a saliva concentration of 70% and stored at −80 °C before experiment^28,36^. Supragingival dental plaque from the buccal surface was collected with a sterile hand probe from 11, 16, 26, 31, 36, 46 teeth of each participant. Samples were deposited into coded sterile plastic tubes with sterile Tris-EDTA buffer solution, transported in ice to the microbiology laboratory and processed within 3 h of sampling. After that, the samples were diluted in sterile glycerol and stored at −80 °C^37^.

### Biofilms using SHI medium

2 mL of each saliva sample was mixed with an equal volume of phosphate-buffered saline (PBS) and mixture was centrifuged at 14,000 g for 3 min. Then, 200 µl supernatant was added to each well that contains one piece of normalized hydroxyapatite(HA) tablet of the 24-well plate to pre-coat the HA tablets and plate were incubated at 37 °C with lid opened for 1 h to dry the saliva coating. Plates were then sterilized under ultraviolet (UV) light for 1 h before 150 µL of each saliva samples were inoculated into pre-coated well containing 850 µL SHI medium. The composition of SHI medium were refer to the previous assay^28^. Plates were incubated at 37 °C under anaerobic conditions to allow biofilm formation.

### 3-[4,5-dimethylthiazol-2-yl]-2,5-diphenyltetrazolium bromide assay of metabolic activity

A 3-[4,5-dimethylthiazol-2-yl]−2,5-diphenyltetrazolium bromide (MTT) assay was used to examine the metabolic activity of the biofilms. MTT is a colorimetric assay that measures the enzymatic reduction of MTT, a yellow tetrazole, to purple formazan. Disks with 2-day biofilms (n = 6) were transferred to a new 24-well plate, and 1 mL of MTT dye (0.5 mg/mL^−1^ MTT in PBS) was added to each well and incubated at 37 °C under 5% CO_2_ for 1 h. During the incubation, metabolically active bacteria metabolized the MTT to formazan inside the living cells. Disks were then transferred to new 24-well plates, and 1 mL of dimethyl sulfoxide (DMSO) was added to solubilize the formazan crystals. The plates were incubated for 20 min with gentle mixing at room temperature. Two hundred microliters of the DMSO solution from each well was collected, and the absorbance was measured at 540 nm using microplate reader (SpectraMax M5; Molecular Devices, Sunnyvale, CA, USA optical density, OD540). The higher absorbance, the higher formazan concentration in wells, indicating higher metabolic activity in the biofilm on the composite surface^10,36^.

### Lactic acid production by biofilms of saliva and plaque of the twins

Disks with 2-day biofilms (n = 6) were rinsed with cysteine peptone water (CPW) to remove loose bacteria, and then transferred to 24-well plates containing 1.5 mL of buffered-peptone water (BPW) plus 0.2% sucrose. The specimens were incubated for 3 h to allow the biofilms to produce acid. Then, the BPW solutions were collected for lactate analysis using an enzymatic method. Absorbance of BPW was determined at 340-nm with a microplate reader (SpectraMax M5; Molecular Devices, Sunnyvale, CA, USA). Standard curves were prepared using a standard lactic acid (Supelco Analytical, Bellefonte, PA, USA) as described in previous studies^10,36^.

### 16S rRNA gene sequencing

The saliva-derived biofilms samples were sent to Shanghai Majorbio Bio-pharm Technology Co., Ltd (Shanghai, China) where the total DNA was extracted, amplified and sequenced according to their standard procedures^38–40^. In brief, microbial DNA was extracted from the saliva-derived biofilms by using the E.Z.N.A.® Soil DNA Kit (Omega Bio-tek) according to manufacturer’s protocols. DNA concentration was assessed by Nanodrop2000 (Thermo Scientific, Wilmington, USA) and DNA quality was determined by 1% agarose gel electrophoresis. The universal target V4–V5 regions of the 16S rRNA gene were PCR-amplified using barcoded primers 515 F (5′-GTGCCAGCMGCCGCGG-3′) and 907 R (5′-CCGTCAATTCMTTTRAGTTT-3′). PCR reactions were performed in triplicate 20 μL mixture containing 4 μL of 5× FastPfu Buffer, 2 μL of 2.5 mM dNTPs, 0.8 μL of each primer (5 μM), 0.4 μL of FastPfu Polymerase and 10 ng of template DNA. The amplicons were then extracted from 2% agarose gels and further purified by using the AxyPrep DNA Gel Extraction Kit (Axygen Biosciences, Union City, CA, USA) and quantified by QuantiFluor -ST (Promega, USA) according to the protocols. Purified amplicons were pooled in equimolar and paired-end sequenced (2 × 300) on an Illumina MiSeq platform (Illumina, San Diego, USA) according to the instruction. The raw reads were deposited into the NCBI Sequence Read Archive (SRA) database (Accession Number: SRP092150).

### Bioinformatics and statistical analysis

Raw reads were demultiplexed and quality-filtered by QIIME (version 1.9.1)^41^. Operational Taxonomic Units (OTUs) were clustered with 98.5% similarity cutoff using UPARSE (version 7.1). The taxonomy of each 16S rRNA gene sequence was analyzed by Ribosomal Database Project Classifier^42^ (http://rdp.cme.msu.edu/) against the Human Oral Microbiome Database with confidence threshold of 70%^43^. Alpha diversity indices (Shannon index, Simpson index)^44^ and richness estimators (ACE index and Chao 1 index)^45^ calculations were performed using Mothur v.1.30.2. Phylogenetic beta diversity measures such as the unweighted UniFrac distance metrics analysis was determined using the represent sequences of OTUs for each sample, and principal component analysis (PCA) was conducted according to the distance matrices. In this study, a P value threshold of 0.05 (Wilcoxon rank-sum test) and an effect size threshold of 2 were used for all biomarkers discussed. OTUs are picked using a closed-reference OTU picking protocol (QIIME 1.9.1) against the Greengenes database pre-clustered at 70% identify. The obtained OTU table was normalized by 16S rRNA copy number. The other data subjected to non-parametric Mann–Whitney analysis or student t test. Differences were considered significant when P < 0.05 and extremely significant when P < 0.01. Software SPSS19.0 (SPSS Inc., Chicago, IL, USA) was applied for statistical analysis.

### Animal experiment

The *in vivo* study was approved by the ethics committee of West China School of Stomatology, Sichuan University (WCHSIRB-D-2016-070), and all experiments were performed according to the National Institutes of Health Guide for the Care and Use of Laboratory Animals.

Germ-free adult male C57BL/6 J mice were maintained in plastic gnotobiotic isolators under a strict 12-hour light cycle, donators ‘saliva were gavage into germ-free recipients (n = 7). Mice were subsequently maintained in separate cages in a gnotobiotic isolator and fed normal diet which was in line with national standard GB-T14924.3-2001. At the 35th day after humanization, mice were killed and saliva samples were collected and stored at −80 °C as soon as possible^30^.

### Data availability

The datasets generated and analyzed during the current study are available from the corresponding author on reasonable requests.

## Electronic supplementary material

Supplementary information

Supplementary Table S1
