# Publisher Correction: Research on oral microbiota of monozygotic twins with discordant caries experience - *in vitro* and *in vivo* study

**DOI:** 10.1038/s41598-020-61785-7

**Published:** 2020-03-10

**Authors:** Hongle Wu, Benhua Zeng, Bolei Li, Biao Ren, Jianhua Zhao, Mingyun Li, Xian Peng, Mingye Feng, Jiyao Li, Hong Wei, Lei Cheng, Xuedong Zhou

**Affiliations:** 1 State Key Laboratory of Oral Diseases, Sichuan University, 610041 Chengdu, China; 2 Department of Cariology and Endodontics, West China Hospital of Stomatology, Sichuan University, Chengdu, 610041 China; 3 National Clinical Research Center for Oral Diseases, Sichuan University, Chengdu, 610041 China; 4 Department of Laboratory Animal Science, College of Basic Medical Sciences, Third Military Medical University, Chongqing, 400038 China; 5 Shanghai Majorbio Bio-pharm Technology Co., Ltd, Shanghai, 201203 China; 6 Department of Immuno-Oncology, Beckman Research Institute, City of Hope Comprehensive Cancer Center, Duarte, CA 91010 USA

Correction to: *Scientific Reports* 10.1038/s41598-018-25636-w, published online 08 May 2018

This Article contains an error in the order of the Figures. Figures 1 and 3 were published as Figures 3 and 1 respectively. The correct Figures 1 and 3 appear below as Figures 1 and 2 respectively. The Figure legends are correct.

**Figure 1:**
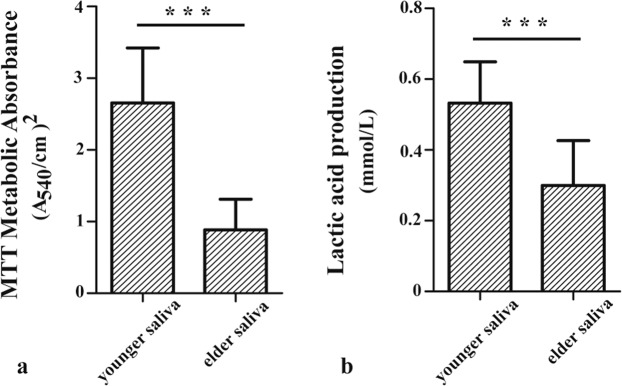
.

**Figure 2:**
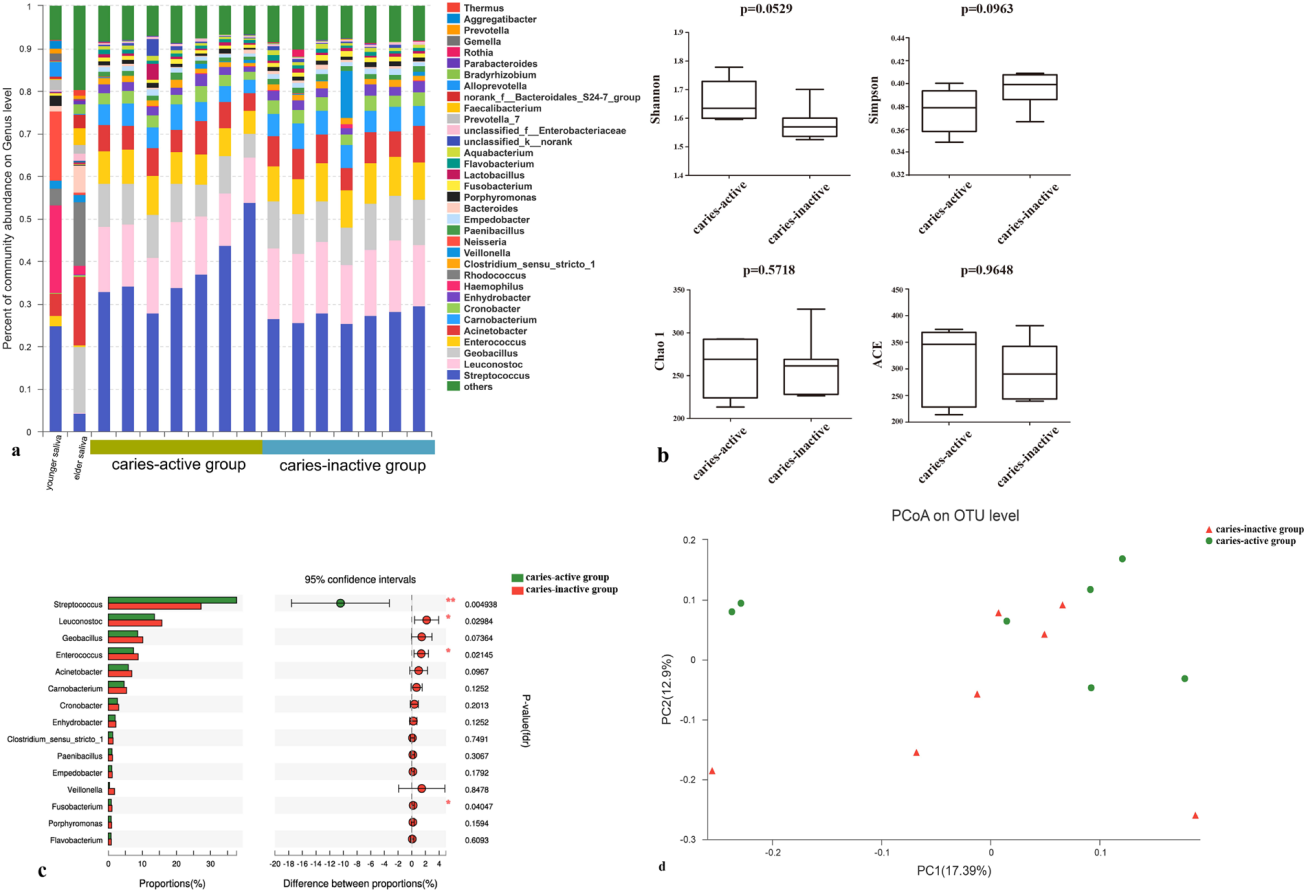
.

In addition, the Acknowledgements section in this Article is incomplete.

“This study was supported by the National Key Research Program of China (2017YFC0840100 and 2017YFC0840107) (L.C.); National Natural Science Foundation of China grant 81600858 (B.R.), and 81430011 (X.Z.); Recruitment Program for Young Professionals (M.F.) the Youth Grant of the Science and Technology Department of Sichuan Province, China 2017JQ0028 (L.C.). Innovative Research Team Program of Sichuan Province (L.C.).”

should read:

“This study was supported by the National Key Research Program of China (2017YFC0840100 and 2017YFC0840107) (L.C.); the Youth Grant of the Science and Technology, Department of Sichuan Province, China 2017JQ0028 (L.C.); National Natural Science Foundation of China grant 81870759 (L.C), 81600858 (B.R.), and 81430011 (X.Z.).”

