# Supplementary material for: Research on oral microbiota of monozygotic twins with discordant caries experience - in vitro and in vivo study
Source: Sci Rep. 2018 May 8;8:7267. doi: 10.1038/s41598-018-25636-w (PMC5940813; doi:10.1038/s41598-018-25636-w)
Supplement: Supplementary file 1 — Supplementary information [file 41598_2018_25636_MOESM1_ESM.pdf]

Supplementary files for

Research on oral microbiota of monozygotic twins with discordant caries experience - *in vitro* and *in vivo* study

Hongle Wu[1,2,3, †], Benhua Zeng[4, †], Bolei Li[1,2,3], Biao Ren[1], Jianhua Zhao[5], Mingyun Li[1], Xian Peng[1], Mingye Feng[1], Jiyao Li[1,2,3], Hong Wei[4, *], Lei Cheng[1,2, ,3, *], and Xuedong Zhou[1,2, ,3, *]

[1] State Key Laboratory of Oral Diseases, Sichuan University, 610041, Chengdu, China.

[2] Dept. of Cariology and Endodontics, West China Hospital of Stomatology, Sichuan University, Chengdu, 610041, China.

[3] National Clinical Research Center for Oral Diseases, Sichuan University, Chengdu, 610041, China.

[4] Department of Laboratory Animal Science, College of Basic Medical Sciences, Third Military Medical University, Chongqing, 400038, China.

[5] Shanghai Majorbio Bio-pharm Technology Co., Ltd, Shanghai, 201203, China;

Patient Consent for Publication in Journals

I give my consent for all or any part of this material to appear in print and online versions of journals under an open access license.
Title of article, photograph, video, or audio:

Research on oral microbiota of monozygotic twins with discordant caries experience - in vitro and in vivo study

____

I understand that:
• My name will not be published with the material and will endeavor to ensure my anonymity. However, I understand that it is possible that somebody, for example members of my family or the health care staff who have looked after me, may recognize me from the image and/or the accompanying text.
• I have reviewed all photographs, personal information form in which I am included that will be published.
•I have known that my donate of saliva and supragingival plaque will only be used in this research.
• Use/re-use of my material may include (without limitation) publication, sharing and adaptation of the material in print and electronic editions of journals, on websites, in sublicensed or reprinted editions (including foreign language editions), and in other works or products if proper accreditation/citation of the original publication is given.
• I can quit at any time but cannot disclose the contents of the experiment to anyone else.

Signed:

____

Date:

____

Print name:

____

If you are not the patient, what is your relationship to him/her?

Witness: __________________________________

Date: ____________________________________

Questionnaire on personal information, lifestyle and dietary habits of twins

1. How often do you brush your teeth every day?
2. When do you brush your teeth?
3. How long do you brush your teeth every time?
4. Is toothpaste used? If is, does the paste contain fluoride?
5. How often do you ingest snacks per day?
6. When do you ingest snacks?
7. Do you eat 3 meals at regular times of the day?
8. Where do you often have your 3 meals?
9. Do you have food before go to bed?
10. When do you get up in the morning and when do you go to bed at night?
11. Do you suffer from allergy to any food or drug?
12. How often do you have beverage?
13. Do you have sweet tongue or not?
14. Do you eat feel that you eat to fast or too slowly?
15. Do you eat all kinds of food or just the food you selected?
16.Did you born in cesarean?

Reference：

1. Taeko K, Hiroki I, et al. Influence of lifestyle on risk of dental caries among children living in urban china[J] Bull Tokyo Dent Coll; 2016;57(3):143-157
2. Carino KMG, Shinada K, et al. Early childhood caries in northern Philippines[J] Community Dent Oral Epidemoil; 2003; 31:81-9

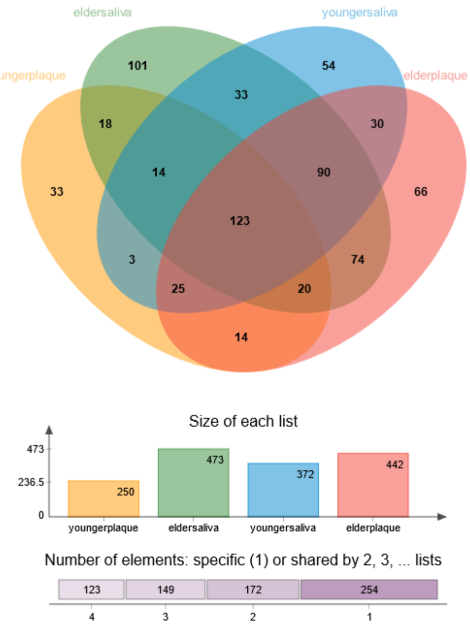

(a)

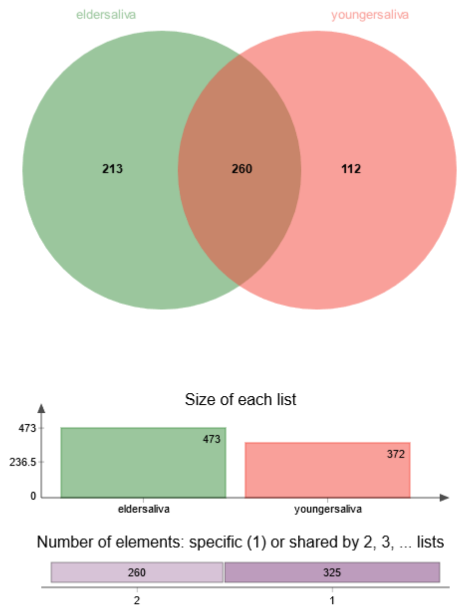

(b)

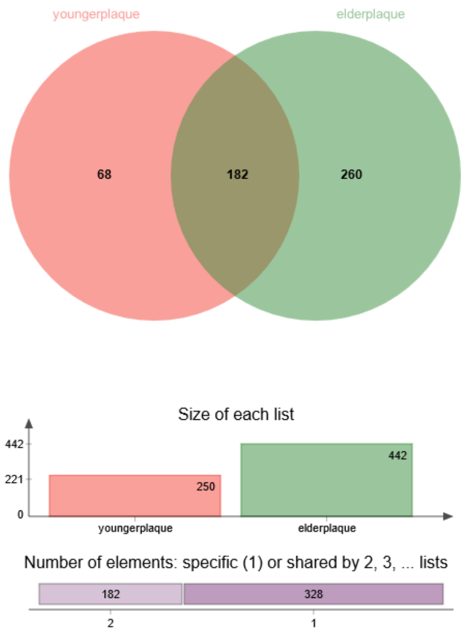

(c)

Figure S1. Venn diagram of the overlap between twins’ plaque and saliva observed operational taxonomic units (OTUs). (a) Venn diagram of the overlap between all 4 samples from host. (b) Venn diagram of the overlap between twins` saliva. (c) Venn diagram of the overlap between twins` plaque. Different color represented different samples. The numbers of singleton OTUs, detected from only one read from one subject, are shown in non-overlap zoom.

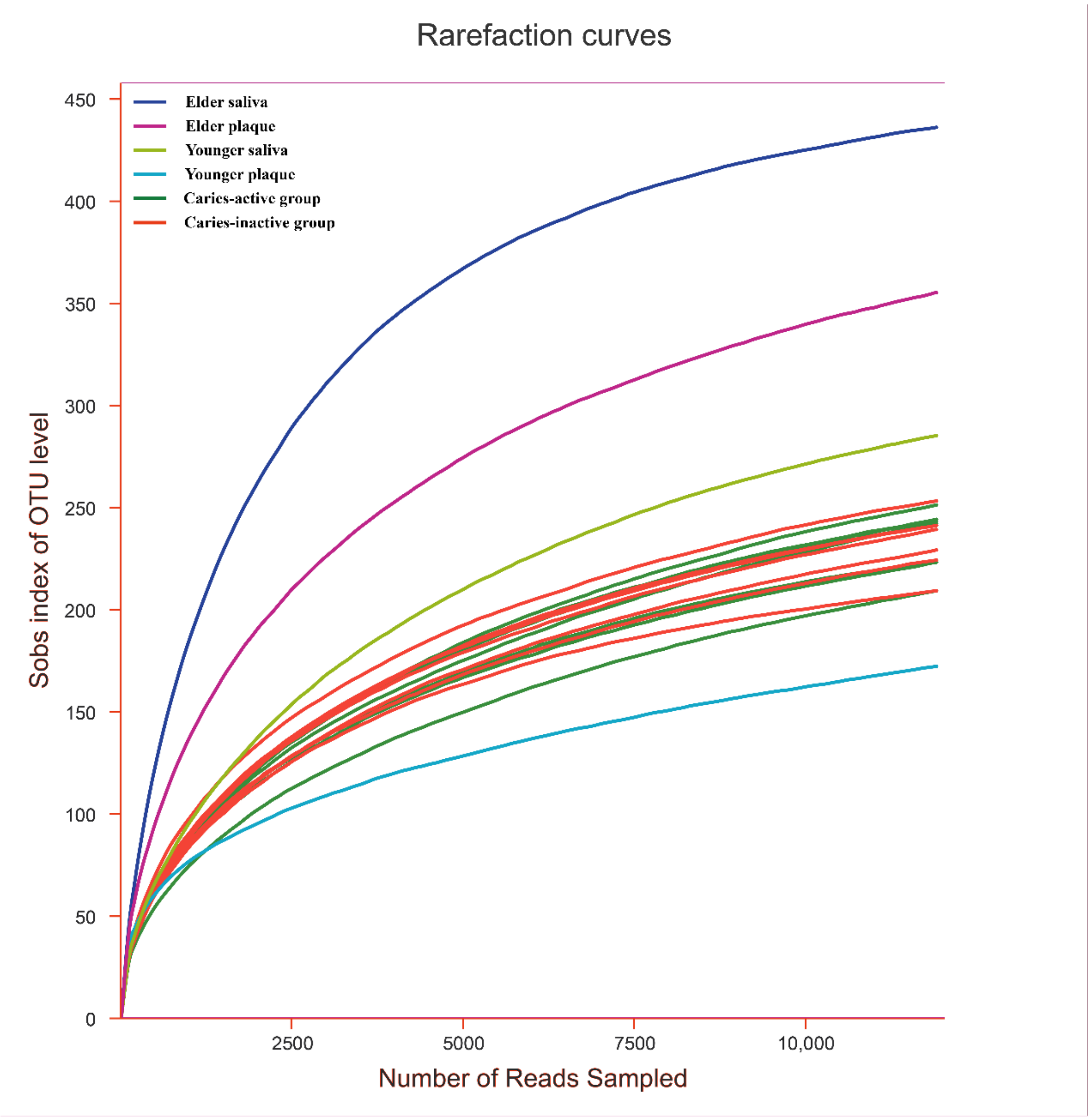

Figure S2. The rarefaction curve of OUT levels for all samples.
